# Clarifying the muddle: towards a comprehensive taxonomy of cognitive biases in medicine

**DOI:** 10.1007/s11019-026-10349-z

**Published:** 2026-04-09

**Authors:** M. Cristina Amoretti, Elisabetta Lalumera

**Affiliations:** 1 DAFIST, Philosophy Section, University of Genoa, Via Balbi 4, 16126 Genoa, Italy; 2 Department for Life Quality Studies (QUVI), University of Bologna, Largo Augusto 237, 47921 Rimini, Italy; 3 The Centre for Philosophy of Epidemiology, Medicine and Public Health, Johannesburg, Republic of South Africa

**Keywords:** Cognitive bias, Diagnostic error, Heuristics, Medical decision-making, Medical reasoning

## Abstract

Cognitive biases are widely discussed in the medical literature as systematic deviations from rational judgment and as significant contributors to diagnostic error. However, the prevailing view of cognitive biases as inherently epistemically detrimental may be challenged by recent research highlighting their potential utility. In this paper, we argue that the impact of cognitive biases in medicine depends not only on their internal cognitive structure, but also on the ecological characteristics of the medical environments in which they operate. We develop a conceptual framework that distinguishes between three types of cognitive biases: (1) those that are epistemically reliable (and practically useful) in so-called “safe cognitive environments”, where ecological informational structures reliably support the use of “fast and frugal” heuristics; (2) those that are practically useful in “pragmatically permeable contexts” such as emergency care or public health, where time pressure, uncertainty, or resource constraints justify decisions that are epistemically suboptimal but action-guiding; and (3) those that are neither epistemically nor pragmatically defensible, and thus constitute clear targets for mitigation. By offering this taxonomy, we aim to clarify when and why cognitive biases hinder or support medical reasoning and practical actions. Our analysis also emphasizes the need to reframe some traditional assumptions in medical epistemology and education. Rather than attempting to eliminate all cognitive biases, we advocate for a more nuanced, context-sensitive, and ecologically informed approach in medicine. We conclude by outlining the implications of this framework for improving diagnostic accuracy, professional training, and institutional practices in healthcare.

## Introduction

Cognitive biases have been increasingly discussed in the medical literature, yet fundamental questions remain unsettled. Are all cognitive biases always epistemically detrimental? Should they always be eliminated or mitigated? Or can they sometimes enhance clinical reasoning and medical decision-making? This paper offers a general conceptual framework to better understand and classify cognitive biases in medicine, aiming to clarify their roles, risks, and potential benefits.

More specifically, we propose a three-part taxonomy that distinguishes between cognitive biases that are (1) epistemically reliable (and practically useful) in so-called “safe cognitive environments”, (2) practically useful in “pragmatically permeable contexts”, though epistemically suboptimal, and (3) neither epistemically nor pragmatically defensible. We argue that in medicine the epistemic and pragmatic impact of cognitive biases depends not only on their intrinsic cognitive mechanisms, but also on the specific ecological features of the medical environments in which they occur.

To develop this view, the paper proceeds as follows. “Cognitive biases in medicine: what and whether” section introduces the concept of cognitive bias as traditionally defined and illustrates its relevance to different areas of medicine. “The bad of cognitive bias” section presents the standard negative view of cognitive biases as systematic causes of error, both in clinical and research settings. “The good of cognitive biases” section challenges this view by highlighting cases in which cognitive biases may prove epistemically and/or pragmatically useful. “A general point on the truth-conduciveness of cognitive biases” and “Are all cognitive biases epistemically and pragmatically equivalent?” sections develop two general arguments—respectively, on the potential truth-conduciveness and pragmatic utility of cognitive biases—emphasizing the importance of cognitive environments and practical constraints. “Tentative conclusions” section introduces our proposed taxonomy and argues that in medicine not all cognitive biases are epistemically and/or pragmatically equivalent. Finally, we conclude by drawing implications for medical education and institutional reform.

## Cognitive biases in medicine: what and whether

Cognitive biases are commonly defined as cognitive processes that systematically deviate from optimal reasoning—where “optimal” is understood in relation to statistical or logical norms of rationality typically employed in decision theory and cognitive psychology—and consequently negatively affect human judgment and decision-making (Gilovich et al. 2002; Gigerenzer and Brighton 2009; Kahneman 2013; Kahneman et al. 1982; Khalidi 2023; Tversky and Kahneman 1974). Typically, cognitive biases are described as predictable (they can be expected to appear under certain circumstances), universal (they affect all individuals regardless of expertise), tenacious (they tend to persist even in those individuals who are aware of them) and unconscious (they are cognitive processes that operate beneath the level of reflective awareness).

Within the domain of medicine, more than one hundred cognitive biases have been identified, influencing not only diagnostic reasoning, but also research practices, treatment planning, prognostic estimations, public health policies, and ethical judgments (Blumenthal-Barby and Krieger 2015; Saposnik et al. 2016). Notably, these biases have been documented across a wide range of medical specialties—from internal (Loncharich et al. 2023) and emergency medicine (Croskerry 2003a, 2003b; Hansen 2020; Kozlovski et al. 2020) to radiology (Branch et al. 2022; Chen et al. 2023; Tee et al. 2022), oncology (Evans et al. 2019; Ozdemir and Finkelstein 2018), obstetrics (Society for Maternal–Fetal Medicine (SMFM) 2002), ophthalmology (Hussain and Oestreicher 2018), pathology (Ko and Glusac 2023), and many others.

One of the most well-documented cognitive biases is anchoring bias, which is the tendency to over rely on some salient features of the initial piece of information (often referred as the “anchor”) and fail to adequately adjust the initial focus when new information becomes available. Closely related is ascertainment bias, in which a doctor’s prior expectations—shaped by stereotypes, past experiences, implicit assumptions or prejudices—skew medical judgment.

Availability bias arises when doctors overestimate (or underestimate) the likelihood of a particular diagnosis or health outcome based on how easily (or difficultly) instances of it come to mind. For example, recent experience with a particular condition (it is more available) may make it seem more probable, leading to overdiagnosis, whereas conditions that have not been encountered recently (they are less available) may be underdiagnosed. Another common cognitive bias is base-rate neglect, where the actual prevalence and/or incidence of a disease is disregarded when interpreting case-specific data, thus resulting in either overestimation or underestimation of its likelihood.

Other forms of cognitive bias relate to the undue weight placed on specific sources of information. Blind obedience, for instance, occurs when doctors overvalue the weight of an authoritative source—such as scientific evidence, laboratory results, imaging, or specialist opinions—which may make it difficult to recognize or act on conflicting findings. Similarly, the bandwagon effect denotes the inclination to align with a diagnosis or decision primarily because it has been adopted by the majority of one’s professional peers. Moreover, confirmation bias is the inclination to seek or prioritize confirming evidence, that is, information that supports a pre-existing hypothesis or diagnosis, while ignoring or downplaying disconfirming evidence.

Another frequent pitfall is expectation bias, which is the tendency to provide outcomes in accordance with what is already expected. Similarly, the framing effect refers to the tendency to draw a conclusion based on how the information is presented, rather than on the information itself. Premature closure is the propensity to terminate the decision-making process prematurely, typically accepting a result before it has been fully verified. In clinical reasoning, it can be summarized by the motto: “when the diagnosis is made, the thinking stops”.

Quite differently, representativeness bias refers to the tendency to make decisions based on how closely a patient’s presentation matches a prototypical case of a disease (prototypical reasoning), which may lead not to consider atypical presentations of a disease and similar but rare conditions. Finally, zebra retreat describes a situation in which a rare but plausible diagnosis (zebra) figures prominently on the differential diagnosis, but it is ultimately abandoned due to various reasons (such as professional insecurity, fear of peer negative judgement, lack of familiarity with the condition, under confidence, fatigue or overwork).

Notably, the biases introduced above are not intended as an exhaustive list of the more than one hundred cognitive biases identified in the literature, but rather as a set of paradigmatic cases widely examined in medicine. More importantly, the cognitive biases we analyse in the following sections were selected not only because they are among the most frequently cited contributors to medical error, but also because they have well-understood underlying heuristics, and display distinct profiles across different environments—making them especially apt for illustrating how our context-sensitive taxonomy operates.

## The bad of cognitive bias

Since cognitive biases are defined as systematic deviations from, or distortions of, optimal reasoning, it may be easily inferred that they ought to be eliminated—or, at least, mitigated—in all medical domains. In fact, many position papers and expert guidelines advocate for the identification and mitigation of cognitive biases, framing them as one of the primary causes of medical error (Croskerry 2003a, 2003b; Graber et al. 2005; Norman et al. 2017; Society for Maternal-Fetal Medicine (SMFM) 2002; Tee et al. 2022; van den Berge and Mamede 2013). In other words, rather than being merely cognitive quirks, biases are seen as systemic contributors to preventable harm, in both medical research and practice.

The influence of cognitive bias is particularly salient in clinical decision-making, where it has been widely associated with diagnostic error (Croskerry 2013; Jala et al. 2023; O’Sullivan and Schofield 2018; Saposnik et al. 2016; Whelehan et al. 2020). The landmark report *To Err Is Human* (Institute of Medicine Committee on Quality of Health Care in America et al. 2000) brought cognitive failures in clinical reasoning to the forefront as a significant cause of harm in healthcare. This concern was deepened in *Improving Diagnosis in Health Care* (Balogh et al. 2015), which identified specific biases—such as anchoring, availability, and premature closure—as key contributors to diagnostic inaccuracies. Medical societies have echoed these findings: the American College of Physicians (ACP) acknowledged the unconscious influence of cognitive processes on clinical judgment (Graber et al. 2005; American College of Physicians 2012), and the American Academy of Family Physicians (AAFP) explicitly addressed cognitive bias as a source of diagnostic failure (American Academy of Family Physicians 2019; Trowbridge et al. 2015). The Society to Improve Diagnosis in Medicine (SIDM), active between 2011 and 2024, consistently highlighted cognitive bias as a central issue in patient safety efforts (Graber et al. 2012; Balogh et al. 2015). Systematic reviews have documented the frequency with which such biases contribute to misdiagnosis, delayed treatment, and avoidable harm (Norman et al. 2017; Monteiro et al. 2020; Graber et al. 2005). A frequently cited estimate suggests that cognitive factors alone may account for up to 28% of diagnostic errors (Balogh et al. 2015), though this figure stems from earlier studies. Notably, these errors typically do not arise from a lack of medical knowledge but from the misapplication of that knowledge through flawed reasoning processes.

The risks of such cognitive errors, however, extend beyond individual clinical encounters. In medical research, for example, biases like bandwagon effect, confirmation bias, and blind obedience can distort the design, interpretation, and dissemination of evidence. For example, Seshia et al. (2016) have shown how a cluster of interrelated biases (“cognitive biases plus”)—among which figure different kinds of cognitive biases—can compromise the objectivity of research programs and evidence hierarchies. Moreover, when replication studies are underfunded or disregarded, and novel findings are favoured due to prestige or perceived authority, epistemic integrity is systematically undermined.

Finally, cognitive biases also pose significant ethical challenges. For example, when clinicians unconsciously apply stereotyping or similar heuristics to cases involving race, gender, age, or disability, they may reinforce disparities and injustices in diagnosis and treatment (Hofmann 2023; Puddifoot 2019, 2021). The ethical dimension of bias-related errors is particularly acute in triage scenarios, palliative care decisions, or end-of-life consultations, in which implicit expectations about patient worth, prognosis, or quality of life can subtly influence clinical judgment. Notably, Katherine Puddifoot’s analysis is primarily concerned with the ethical evaluation of stereotyping in healthcare, especially insofar as it contributes to forms of epistemic injustice, such as credibility deficits and failures to recognize patients as epistemic agents. A central insight of her work is that even epistemically accurate stereotyping may remain ethically problematic in clinical contexts marked by asymmetric power relations. While we fully acknowledge the importance of this ethical perspective, our aim in this paper is different. We bracket questions of ethical permissibility and epistemic injustice in order to focus on how the epistemic and pragmatic status of cognitive biases and heuristics in medicine varies with the structure of the cognitive environment and the aims of medical reasoning.

## The good of cognitive biases

While most position papers recommend the elimination or mitigation of cognitive biases, some noteworthy contributions have already challenged this predominantly negative view by exploring the potential benefits of certain cognitive biases in specific contexts (Lieder et al. 2017; Mangus and Mahajan 2022; Marewski and Gigerenzer 2012; Monteiro et al. 2020). Indeed, an open and increasingly relevant question concerns whether, in some circumstances, these so-called cognitive biases might instead yield beneficial epistemic and/or pragmatic outcomes in medicine (Whelehan et al. 2020).

In fact, far from being mere flaws in rationality, cognitive biases can be regarded as “markers or signatures” of an underlying heuristic (Gilovich et al. 2002), where “fast and frugal” heuristics (Gigerenzer and Brighton 2009) are mental shortcuts that enable clinicians to make epistemically reliable and pragmatically effective decisions, especially under conditions of uncertainty, limited information, and time constraints. In what follows, we examine three illustrative cases in which cognitive biases reveal an underlying heuristic, proving to be both epistemically and pragmatically beneficial, enhancing doctor performance and improving patient health outcomes.

The first epistemic and pragmatic advantage of cognitive biases involves fast reasoning. In emergency medicine, for example, quick decisions are often a matter of life and death, and here cognitive bias can often help (Croskerry 2002). Let’s consider, for example, the representativeness bias, that is the tendency to make decisions based on how closely a condition matches a prototypical case (prototypical reasoning). Now imagine a high-pressure environment, such as an emergency department, where rapid decision-making is critical. If a patient presents with prototypical symptoms of a condition like acute appendicitis—that is, right lower quadrant pain, nausea, vomiting, and rebound and McBurney’s point tenderness—the representativeness bias can enable faster decision-making and guide the physician toward a reliable diagnosis, drastically reducing time to intervention and possible negative outcomes. While this may not be as thorough as an extensive differential diagnosis, in urgent situations, the speed of decision-making can be crucial for timely intervention. Thus, although prototypical reasoning can sometimes lead to diagnostic errors—especially when atypical presentations are involved or rare conditions mimic common ones—it can also be highly useful in situations where time and knowledge are limited. When a patient’s presentation closely matches a well-established pattern of symptoms, categorizing patients into diagnostic groups based on their resemblance to such known prototypes usually leads to a reliable and prompt diagnosis (Amoretti et al. 2017). In this case, representativeness bias is both truth conducive and pragmatically useful.

Second, cognitive biases can also be advantageous in reducing cognitive workload. Consider, for instance, confirmation bias, broadly understood as the tendency to seek or prioritize information that supports an initial hypothesis, rather than actively look for disconfirming evidence. In this wider sense, confirmation bias simply refers to a confirmatory orientation in information gathering, without necessarily involving selective seeking of evidence or the ignoring of falsifying cues. Now imagine a physician trying to determine whether a patient should be assigned to the coronary care unit (CCU) or to a regular nursing bed (Marewski and Gigerenzer 2012). The physician knows that if the patient has a myocardial infarction, then an anomaly in the patient’s electrocardiogram (such as an ST-segment elevation) is detected. So, if such an anomaly is detected, then the patient is immediately assigned to the CCU. This initial step is designed to quickly confirm the most likely diagnosis—myocardial infarction (heart attack)—based on a highly reliable indicator (ST-segment elevation). The presence of this anomaly “confirms” the clinician’s hypothesis that the patient is experiencing a cardiac event, and the decision to admit the patient to the CCU follows swiftly. If this first criterion is not met, the physician moves on to a second one, chest pain, and, if neither of the first two indicators is met, they ask a third composite question, which considers five other predictors of myocardial infarction. At each step, the clinician is then seeking to “confirm” the diagnosis and consequently decide to admit the patient to the CCU. In this scenario, confirmation bias helps to reduce the cognitive burden on the physician by focusing on sequentially confirming the likelihood of a cardiac event through a series of increasingly specific predictors before moving on to consider less likely alternatives. Though confirmation bias can sometimes lead to errors by overlooking alternative diagnoses, in this context, it reveals to be useful by prioritizing the identification of life-threatening conditions, such as myocardial infarction, without the need of complex and cognitive demanding reasoning. Again, confirmation bias is both truth conducive and pragmatically useful.

Third, radiology illustrates how cognitive biases, rather than being mere flaws, often form the very scaffolding of expert interpretation (Itri and Patel 2018). In fact, the discipline heavily relies on pattern recognition, allowing radiologists to make fast, high-volume decisions based on familiarity with visual prototypes. While such processes are frequently flagged as vulnerable to bias (Chen et al. 2023)—such as premature closure or representativeness—they are also essential for skilled performance. A case in point: a PET/CT scan reveals tracer uptake in the left axillary and supraclavicular lymph nodes. Based on the prototypical pattern, an experienced radiologist might suspect metastatic disease. However, knowledge of the patient’s recent COVID-19 vaccination on the same side shifts the interpretation toward a benign inflammatory response. Of course, this contextual cue does not allow the radiologist to *decisively* conclude that the uptake is vaccine-related; rather, it introduces a plausible competing hypothesis that must be further evaluated and, if needed, confirmed or disconfirmed through additional diagnostic steps. What might have been representativeness bias becomes, instead, a demonstration of how contextual sensitivity—integrating non-imaging data—refines heuristic reasoning. This kind of expertise, shaped by experience and environmental attunement, cannot be fully captured by strict rule-following or algorithmic precision (Johnson et al. 2021). This case exemplifies a broader epistemological point: what appears as cognitive bias from the outside may be instead an interpretive resource from within. Radiological expertise blends the rapid application of prototypical schemas with responsiveness to patient-specific cues. Cognitive shortcuts, in this setting, support both efficiency and accuracy. Of course, this does not mean that such expert heuristics deliver certainty, but that they are epistemically reliable and truth-conducive: they tend to steer interpretation toward the correct diagnosis within an appropriate cognitive environment. Debiasing, in this sense, may strip expert cognition of its core strength. In fact, speaking of machine learning (ML) in radiology, it is well-known that systems designed to reduce cognitive bias can produce unintended consequences: while they improve the performance of novice readers, they often impair that of experts by disrupting the pattern recognition processes essential to skilled diagnosis. These distortions can then be encoded into ML training data, perpetuating structural inequities in diagnostic algorithms (Povyakalo et al. 2013). Even more provocatively, Pot et al. (2021) highlights how in radiology certain cognitive biases may serve corrective or justice-oriented purposes. For example, consciously adjusting image interpretations to avoid underdiagnosing marginalized groups can introduce a form of equitable bias. In this light, cognitive bias becomes not just a deviation from a neutral standard, but a tool for addressing epistemic injustice. Altogether, these insights suggest a reframing of cognitive bias in radiology. Rather than treating cognitive bias solely as a deviation from objectivity, we should understand it as a complex, context-dependent feature of human reasoning. Cognitive bias may reflect the very mechanisms that enable expertise, as well as the social responsibilities embedded in clinical judgment. The aim, then, is not wholesale elimination, but critical discernment: to distinguish when cognitive bias hinders or harms—and when it helps, adapts, or repairs.

## A general point on the truth-conduciveness of cognitive biases

To begin with, what appears to be a cognitive bias under an abstract, context-independent statistical or logical standard can reveal itself to be *epistemically reliable and truth-conducive* once the relevant environmental conditions are specified and granted. To put it differently, certain cognitive processes traditionally regarded as biases—as they involve a systematic deviation from the norms of rationality—can be better understood as context-sensitive reliable *heuristics*—that is, as mental shortcuts that may, or may not, yield the normatively correct outcome (statistically or logically), depending on the context of their application. This differentiation agrees with standard usage in cognitive science (Gilovich et al. 2002; Gigerenzer and Brighton 2009; Kahneman 2013), where heuristics are typically regarded as mental processes that may, or may not, result in an error (statistically or logically speaking), but which nevertheless are epistemically reliable and truth-conducive in what we call “*safe* cognitive environments”*.* These environments are contexts characterized by relatively stable statistical regularities and low ambiguity, where certain types of errors are unlikely to occur (Gigerenzer et al. 1999; Gigerenzer and Gaissmaier 2011; Todd and Gigerenzer 2000, 2012).

For instance, anchoring may not always be epistemically detrimental. If the initial “anchor” is itself reliable—such as a highly specific symptom or laboratory value—then relying on it may streamline diagnostic reasoning in an epistemically reliable way. Recent experimental studies suggest that anchoring may reflect not a cognitive failure but an adaptive and epistemically reliable use of limited cognitive resources (Lieder et al. 2017). Similarly, framing effect can be epistemically benign when the frame derives from a clinically sound anamnesis or from appropriately contextualized information. Availability bias also exhibits this dual nature. When recent experience aligns with the epidemiological prevalence of a condition, availability-based reasoning may increase diagnostic reliability. For example, during seasonal flu epidemics or outbreaks of infectious diseases, the tendency to overestimate the likelihood of a recently encountered diagnosis may correspond closely to actual incidence data. In such cases, the clinician’s experiential familiarity with a disease becomes a form of embodied knowledge that operates epistemically reliably under limited knowledge and time constraints.

What is particularly relevant for our purposes is that the epistemic success of these heuristic strategies presupposes a set of environmental conditions that closely align with what we call *safe* cognitive environments. This point is made explicit in the medical education literature on clinical reasoning. For example, Hassan (2013) emphasizes that heuristic and pattern-recognition strategies—though acknowledged to be less fine-grained and more error-prone than high-fidelity visual pattern recognition—are routinely and legitimately employed by expert clinicians in acute diagnostic contexts. According to Hassan, such heuristics perform well when clinical presentations follow familiar patterns, when informational inputs such as history-taking, physical examination, and diagnostic tests are reliable, and when institutional scaffolding—such as guidelines, decision maps, and structured cognitive aids—reduces noise and ambiguity in decision-making. Under these conditions, heuristic reasoning does not function as a systematic source of error, but rather as an epistemically reliable way of exploiting stable regularities in the clinical environment. This medical literature thus provides independent support for our claim that the truth-conduciveness of cognitive processes traditionally labelled as biases cannot be assessed in abstraction from the environments in which they operate.

A further epistemic feature of safe cognitive environments concerns the robustness of heuristic strategies to noise and informational redundancy. Medical education research has emphasized that, in uncertain clinical domains, reasoning strategies that incorporate large amounts of information may be more vulnerable to tracking spurious or locally specific correlations, thereby undermining their reliability across cases. By contrast, simple heuristic strategies that deliberately ignore part of the available information and rely on a small number of well-established cues may generalize better across patients and contexts, precisely because they are less sensitive to noise and redundant inputs (Wegwarth et al. 2009). From this perspective, the epistemic value of heuristics does not lie in approximating an ideal standard of optimization, but in their capacity to remain reliable across non-ideal conditions—an aspect that contributes to their truth-conduciveness in safe cognitive environments.

Strictly speaking, in safe cognitive environments, cognitive biases that can be better understood as context-sensitive reliable heuristics no longer fall under the very definition of cognitive bias: they are epistemically reliable and truth-conducive insofar as they track appropriate ecological cues that support their use. This means, however, that their truth-conduciveness depends heavily on the degree of “ecological fit” between the heuristic strategy and the medical setting.

This environment-relative approach is also supported by recent work in cognitive ontology. On this respect, Khalidi (2023) argues that neither “heuristics” nor “biases” constitute homogeneous cognitive kinds, and that their classification cannot be fixed independently of the tasks and environments in which they are deployed. On this view, whether a cognitive process counts as a heuristic or as a cognitive bias depends not only on its internal features, but also on how it interacts with the informational structure of the environment and the aims of the task. This taxonomic perspective reinforces our claim that the epistemic status of cognitive biases in medicine cannot be assessed in abstraction from their deployment conditions—as we will see in more details in the next section.

More generally, our framework allows us to specify at least three criteria that delineate what counts as a *safe* cognitive environment in medical decision-making. First, *safe* cognitive environments exhibit stable and predictable statistical regularities, such as well-understood symptom clusters or established disease prevalences, which enable clinicians to apply heuristics in a truth-conducive manner. Second, they are characterized by low levels of noise, ambiguity, or informational distortion: laboratory tests and imaging devices function correctly, clinical records are accurate and complete, and patients provide truthful and relevant information. Third, *safe* cognitive environments involve a low likelihood of encountering atypical, rare, or unprecedented clinical presentations, ensuring that clinicians’ expectations, diagnostic schemas, and the structure of the environment remain sufficiently aligned. When one or more of these conditions are violated, the cognitive environment becomes *unsafe*, systematically undermining the truth conduciveness and epistemic reliability of context-sensitive heuristic shortcuts.

To better understand when and why cognitive biases lead to diagnostic errors, it is thus essential to consider not only cognitive mechanisms by themselves, but also the external features of the environmental situations in which these mechanisms operate. In this regard, the distinction between *safe* and *unsafe* cognitive environments proves particularly useful. As we have seen, *safe* cognitive environments are contexts in which heuristics underlying cognitive biases are truth-conducive, yielding epistemically reliable results. Conversely, *unsafe* cognitive environments are those characterized by contextual or structural features—such as a high probability of error, atypical presentations, or misleading contextual cues—which systematically increase the risks of epistemic unreliability and false results.

*Unsafe* cognitive environments can be compared with a well-known class of thought experiments in epistemology, often referred to as “fake barn scenarios” (Goldman 1976; Sosa 2007). In such cases, an agent relies on perceptual or inferential mechanisms that are generally truth-conducive, but which are rendered unreliable by deceptive features of the environment. For instance, a person driving through a countryside filled with façades that only look like barns—except for one real barn which is the one they are actually looking at—may form the belief “that is a barn” based on accurate perception, yet still fail to possess genuine knowledge because the environment is epistemically deceitful. In this case, the agent’s belief-forming process is not faulty per se, but the setting is engineered in such a way that systematically increases the risks of false and unreliable results. A striking illustration of how *unsafe* cognitive environments can compromise otherwise reliable cognitive mechanisms is provided by the well-known study by David Rosenhan (1973), in which eight “pseudo patients” (including Rosenhan himself) feigned psychiatric symptoms to gain admission to mental hospitals. Despite clinicians relying on reliable diagnostic criteria, the deception embedded in the environment—that is, the fake psychiatric symptoms—led to systematic misjudgements.

The notion of *unsafe* cognitive environments also bears a strong affinity with *hostile environments*, a concept developed by Sterelny (2003, 2010) in the context of philosophy of mind and biology. Sterelny distinguishes between epistemically “friendly” environments, where agents can reliably acquire true beliefs with minimal cognitive support, and epistemically hostile environments, which are characterized by deception, informational asymmetry, social manipulation, or structural opacity. The parallel is instructive: just as Sterelny’s hostile environments undermine reliable belief formation, unsafe cognitive environments in medicine compromise the conditions for reliable diagnostic and clinical reasoning.

In contemporary medical practice there are many examples of *unsafe* cognitive environments. First, unprecedented clinical situations, such as the early cases of COVID-19 or AIDS, disrupted diagnostic expectations because clinicians attempted to fit specific symptom constellations into familiar diagnostic categories (e.g., interpreting COVID-19 as influenza, or early AIDS as pneumonia). Second, patient deception or non-cooperation, such as withholding critical health information due to stigma or fear, can create misleading clinical pictures. Third, organizational, technical, or equipment failures (e.g., laboratory errors, malfunctioning imaging devices) may prompt clinicians to form their judgment based on flawed or mistaken data. Fourth, chronic work overload, fatigue, and sleep deprivation—well-documented features of many healthcare systems—can exacerbate the use of cognitive shortcuts and reduce the capacity for reflective reasoning.

Such *unsafe* cognitive environments challenge the traditional assumption that individual practitioners are the sole locus of epistemic responsibility and the related idea that diagnostic failures are to be attributed to the presence of cognitive biases per se. Instead, errors emerge from the interaction between some cognitive processes and unsafe cognitive environments, which distort the informational inputs upon which context-sensitive reliable heuristics typically rely. This perspective thus invites a shift in framing cognitive bias in medicine. From the idea that cognitive biases cause errors in medical judgment (*sic et simpliciter*), to the recognition that cognitive biases cause errors in medical judgment *when the underlying context-sensitive heuristic is deployed in unsafe cognitive environments*. Rather than focusing exclusively on debiasing the individual—through training, metacognitive awareness, or cognitive forcing strategies—this approach suggests the need for also redesigning clinical environments to support more robust reasoning.

It also invites a further nuance. Not all *unsafe* environments are alike. We may distinguish between *extraordinary* unsafe environments, such as the onset of a novel pandemic, which are difficult to anticipate or prevent, and *ordinary* unsafe environments, such as those marked by poor doctor-patient communication, systemic understaffing or time pressure, which have detectable educational and structural causes that may be differently addressed and mitigated.

## A general point on the pragmatic utility of cognitive biases

In the previous section, we have shown that what appears to be a cognitive bias under an abstract, context-independent statistical or logical standard can be better understood as a *context-sensitive heuristic*, which is epistemically reliable and truth-conducive when deployed in *safe* cognitive environments. However, a further—and perhaps more provocative—point emerges when we shift the focus from epistemic reliability and truth-conduciveness to *pragmatic utility*. In certain real-world circumstances, judgements that would be considered wrong according to statistical or logical normative standards may nonetheless prove pragmatically valuable. In other words, in what we call *pragmatically permeable contexts*, cognitive biases cannot be understood as context-sensitive reliable heuristics, as in the previous case; yet—even while actually involving a deviation from the norms of rationality—they can still be *beneficial and practically valuable* cognitive processes by improving clinical care. In fact, in *pragmatically permeable* contexts, the aims of medical decision-making are not exclusively epistemic: promoting patient well-being may legitimately take precedence over strict truth-conduciveness.

Imagine walking through a forest and being told that a creature known as the Gruffalo inhabits the area. It is described as having coarse, shaggy fur, long and sharp teeth, an enormous mouth with powerful jaws, long and pointed claws, and large glowing eyes. Based on this description, the inference that the Gruffalo is a ferocious and dangerous mammal is statistically less probable than the simpler inference that the Gruffalo is merely a mammal. This is because, from a probabilistic perspective, the likelihood that an entity belongs to both category M (mammal) and category F (ferocious)—P(M&F)—is always lower than the likelihood that it belongs to M alone—P(M). However, if you suddenly hear someone shout “There’s the Gruffalo!”, making the former inference—based on representativeness—may prove more pragmatically useful than the latter, despite being wrong in a strict probabilistic sense. In fact, it supports effective and timely action, such as fleeing the scene, which could ultimately prove lifesaving (Amoretti et al. 2017; Frixione and Lieto 2014). Moreover, even if it should later turn out that the Gruffalo is in fact a nice and gentle mammal—thus rendering the inference not only incorrect from a probabilistic perspective but also factually false—the conclusion would still remain pragmatically useful, given that fleeing the scene poses minimal risk, and incurs no significant cost. To sum up, a judgement based on representativeness—though probabilistically incorrect and even later revealed factually false—may nonetheless prove pragmatically preferable than the correct judgement based on likelihood.

A similar logic can also apply in clinical medicine and public-health settings, where ethical and practical constraints—such as time pressure, limited knowledge and resources, or the imperative to cure people and minimize harm—can be different and more stringent than in the context of medical research.

Consider, for example, a physician confronted with a patient exhibiting ear pain and fever persisting for two days. Given that bacterial otitis media typically entails these symptoms (i.e., *if* bacterial otitis, *then* pain and fever), the presence of such symptoms may be interpreted as confirming the diagnosis (pain and fever, *therefore* bacterial otitis). While this inference is incorrect from the point of view of classical logic—specifically, it is a form of confirmation bias—it may nonetheless be pragmatically useful, even if it ultimately turns out that the otitis is not bacterial. In a context where knowledge and diagnostic tools are limited, or where swift intervention is necessary, initiating empiric treatment with a broad-spectrum antibiotic may represent the most reasonable and beneficial course of action. Such a response not only avoids the risks associated with delayed treatment of a potentially serious condition, but also reduces the need for further testing—tests which may be invasive, expensive, or unavailable. In this way, the use of a fallacious but action-guiding inference becomes justified on pragmatic grounds, as long as the potential downsides of overtreatment are minimal and the potential benefits substantial.

Such examples highlight the importance of what we call *pragmatically permeable contexts*, that is, domains like clinical medicine and public health settings where ethical, practical, and institutional constraints constitutively shape the conditions of decision-making. In these contexts, the primary goal is not always truth or epistemic reliability, but rather timely and effective interventions able to maximize beneficial pragmatic outcomes, such as harm reduction, cost-effectiveness, or rapid triage.

This perspective suggests a further refinement in how we understand the relationship between cognitive biases and medical error. From the idea that cognitive biases cause errors in medical judgments (*sic et simpliciter*), to the more nuanced indication that cognitive biases may cause errors in medical judgments (according to normative rational theory), *but in pragmatically permeable contexts, such errors may still have practical utility*, generating actions that are useful, efficient, and ethically justifiable.

This shift has important implications. It invites us to distinguish between truth-oriented and action-oriented reasoning and to evaluate the role of cognitive biases not solely by their adherence to logical standards but also by their context-sensitive utility. Recognizing this dual dimension—epistemic and pragmatic—opens the door to a more flexible and realistic account of clinical decision-making, one that acknowledges the trade-offs clinicians must navigate in real-world environments.

## Are all cognitive biases epistemically and pragmatically equivalent?

The preceding sections have shown that, under certain conditions, cognitive biases may function as context-sensitive reliable heuristics or *beneficial and practically valuable* cognitive processes—either because they lead to accurate judgments in *safe cognitive environments*, or because they support effective actions in *pragmatically permeable contexts*. However, an important question arises: do all cognitive biases have the potential to be rehabilitated in this way? That is, given the appropriate environment, can any cognitive bias be reframed as epistemically and/or pragmatically beneficial?

We suggest that the answer to these questions is *no*. Cognitive biases are not all of the same kind—as convincingly argued elsewhere (Kahlidi 2023)—nor do they all respond similarly to contextual cues. Their epistemic and/or pragmatic value varies depending on the nature of the bias, the structure of the environment, and the goals of reasoning in a given domain. Below, we propose a tentative three-part classification that—without purporting to identify natural sub-kinds of cognitive bias, or trying to reveal any kindhood among their underlying heuristics (for a wider discussion on this topic, see Kahlidi 2023)—distinguishes cognitive biases on the basis of their potential epistemic and/or pragmatic utility, that is their capacity to produce epistemically reliable and/or action-guiding outcomes in specific environments. Importantly, this taxonomy is context-dependent rather than fixed: it does not rule out possible overlaps, in that the same cognitive process may fall into different categories depending on environmental features and decision-making goals. For instance, representativeness can be truth-conducive in a safe cognitive environment (category 1 below) yet only pragmatically useful—and epistemically unreliable—in a pragmatically permeable context (category 2 below). The examples offered here are illustrative and not meant to be exhaustive.

1. Cognitive strategies that may, or may not, yield the normatively correct outcome (statistically or logically), but which nevertheless are epistemically reliable and truth-conducive in *safe cognitive environments.* This first category includes cognitive processes that are typically regarded as cognitive biases under an abstract, context-independent statistical or logical standard, but that can be better understood as context-sensitive heuristics that are epistemically reliable and truth conducive when applied in specific environmental conditions. As we have seen, anchoring bias may be epistemically reliable when the anchor is precise, relevant, and statistically justified; framing effect may be epistemically beneficial when the frame is constructed from an accurate anamnesis or clinical context; availability bias may be truth-conducive when the clinician’s expectations are shaped by extensive experience in a given population or epidemiological setting; representativeness bias may be epistemically reliable when a strong and well-established correlation exists between phenotype and disease (e.g., the classic “Parkinsonian gait” or the “thyrotoxic appearance” of hyperthyroidism). In all these cases, cognitive biases are better understood in terms of their underlying “fast and frugal” heuristics that exploit environmental regularities to reduce cognitive effort and time without compromising epistemic reliability and truth-conduciveness. Notably, context-sensitive reliable heuristics are typically practically efficient as well, thereby aligning epistemic reliability and truth conduciveness with pragmatic usefulness.
2. Cognitive strategies that identify the most pragmatically useful outcome, even if such an outcome may not be the right or the optimal result, as established by either statistical or logical normative frameworks. This second category encompasses cognitive biases that, in pragmatically permeable contexts, prove to be practically useful, though epistemically suboptimal, since they may actually lead to logically or statistically wrong conclusions. In fact, in pragmatically permeable contexts—such as clinical medicine or public-health settings—the cost of error is not uniformly distributed: acting quickly on a likely (even if false) hypothesis may save lives or prevent harm, whereas epistemic thoroughness may be inefficient or even dangerous for people. As we have seen, representativeness bias and confirmation bias can be pragmatically useful even when they actually lead to a wrong conclusion. Acting according to prototypical reasoning and looking for confirmations may prove to be practical preferable compared to overutilization of diagnostic tests to exclude all conceivable diagnoses—especially when immediate intervention is needed and its potential benefits substantial, while the risks associated with hypothetical overtreatment are minimal. Premature closure may be pragmatically useful too, when the suspected diagnosis already warrants urgent action (for example, suspected myocardial infarction or peritonitis), or when the proposed treatment has few adverse effects and a high safety margin (for example, administering broad-spectrum antibiotics in the presence of ear pain and fever). In all these pragmatically permeable contexts, the pragmatic imperative to act may outweigh the epistemic imperative to aim at truth. Cognitive biases may thus support instrumentally rational decisions, especially when they minimize harm or maximize benefit under limited knowledge and time constraints. To put it another way, in pragmatically permeable contexts, pragmatic utility may legitimately take precedence over strict truth-conduciveness, making some cognitive biases instrumentally rational despite their epistemic limitations.
3. Cognitive processes that systematically deviate from the right result, as established by either statistical or logical normative frameworks, without having any pragmatic utility as well. This third category—which encompasses biases that are neither epistemically reliable nor pragmatically useful—somehow matches with the traditional general definition of cognitive biases. In fact, it includes cognitive processes that systematically generate erroneous conclusions regardless of context and offer no compensatory benefit in terms of action or practical efficiency. Cognitive biases that fall in this category may be base-rate neglect, overconfidence (Berner and Graber 2008), and blind obedience. Notably, all these biases tend to be constitutively associated with unsafe cognitive environments, characterized by stress, cognitive overload, systemic inefficiencies, or faulty informational inputs. Unlike the previous two categories, these cognitive biases neither exploit environmental regularities to be truth-conducive, nor support effective action. As such, they actively undermine both epistemic and pragmatic goals, thus remaining clear targets for mitigation strategies.

As already noted, Khalidi’s discussion of the *confirmation bias* (Kahalidi 2023, pp. 199–208) illustrates precisely the kind of context-sensitivity that our taxonomy aims to capture. As he argues, what counts as a *confirmation bias* cannot be identified solely by inspecting the cognitive mechanism in isolation. The same underlying heuristic may be adaptive and even epistemically reliable and truth-conducive in some environments, while generating systematic error in others; and whether a given instance should be classified as a bias depends on features external to the thinker, such as the task structure, the social setting, the agent’s expertise, or the justification of their prior beliefs. Similar reflections can be found in Puddifoot’s analysis of stereotyping (Puddifoot 2019, 2021). She argues that the epistemic impact of stereotyping does not derive from the cognitive process per se, but from how automatic associations operate under specific contextual pressures—such as high workload, time constraints, stress, or asymmetric doctor-patient interactions. In this sense, both stereotyping and the heuristics underlying cognitive biases are relational phenomena: their epistemic status is determined by features of the broader cognitive and social environment, not by their internal structure alone. These considerations reinforce our central claim: in medicine, cognitive bias cannot be addressed independently of the contexts in which they operate, and the line between a beneficial heuristic and a problematic bias is often relational rather than intrinsic.

At the same time, our taxonomy extends Khalidi’s and Puddifoot’s analysis by highlighting not only the epistemic dimension of this context-sensitivity, but also its pragmatic dimension. In medicine especially, practical goals—such as minimizing harm, stabilising a patient, or enabling timely intervention—may at times legitimately outweigh the epistemic aim of tracking the truth. As a result, a cognitive process may fail to be truth-conducive while nevertheless being pragmatically valuable within a particular context—that is, a pragmatically permeable context. Recognizing this possible disjunction between epistemic and pragmatic utility is crucial for evaluating the impact of cognitive biases in real-world medical practice.

In addition, we would like to clarify more explicitly how our analysis differs from and complements Puddifoot’s work. Puddifoot’s discussion of stereotyping in medicine is primarily motivated by the ethical evaluation of biased cognition in contexts of asymmetric power relations, and is explicitly framed through the lens of epistemic injustice. Even when stereotyping leads to epistemically accurate judgments, Puddifoot argues that it may remain ethically problematic insofar as it undermines patients’ credibility or fails to treat them as epistemic agents. By contrast, our analysis deliberately brackets questions of ethical permissibility and epistemic injustice in order to focus on a different problem: how the epistemic and pragmatic status of cognitive biases and heuristics in medical reasoning varies with the structure of the cognitive environment and with the aims of clinical practice. This shift in focus allows us to develop a general taxonomy of cognitive biases in medicine—extending beyond the sole stereotyping—and to distinguish systematically between epistemically reliable heuristics, pragmatically valuable but epistemically suboptimal biases, and genuinely problematic biases. We take this framework to be complementary to, rather than a restatement of, Puddifoot’s analysis.

To conclude, our taxonomy converges with and expands upon other contemporary views by emphasising that efforts to understand and address cognitive bias must be adequately differentiated, context-aware, and goal-sensitive. Rather than treating all cognitive biases as equally problematic, we should ask, in each specific context, whether the cognitive process in question supports reliable knowledge, useful action, neither—or both.

## Tentative conclusions

In this paper, we argued that cognitive biases in medicine are not inherently detrimental; rather, their epistemic and pragmatic value depends on the contexts in which they operate. Some cognitive biases, when functioning as context-sensitive reliable heuristics within *safe cognitive environments*, are epistemically reliable and truth conducive, and thereby promote both epistemic and practical goals; others, when operating within *pragmatically permeable contexts*—such as clinical medicine and public health settings—may enhance pragmatic effectiveness even while deviating from the norms of rationality. This insight invites a conceptual and practical shift in medicine: rather than attempting to identify and eliminate *all* cognitive biases indiscriminately, we should adopt a more nuanced approach, which distinguishes between different types of cognitive biases, recognizes the specific features of the environments in which they arise and operate, and weights their potential epistemic and pragmatic benefits alongside their risks.

This ecological perspective has important implications for medical education and health policy. Traditional efforts to “debias” clinicians often underestimate the complexity of medical reasoning in real-world scenarios. Debiasing is rarely straightforward: no single strategy is effective across all cognitive biases, interventions often need to be tailored to specific cases and environments, and long-term reinforcement is likely required.

First, not all cognitive biases work in the same way across different contexts. Thus, rather than treating them as uniform cognitive flaws, we should investigate how different cognitive biases interact with specific environmental conditions to produce specific outcomes. In particular, it is important to understand how the truth-conduciveness of certain cognitive biases strongly depends on safe cognitive environments—that is, settings that are stable, informative, and trustworthy.

Second, some medical errors that are traditionally attributed to faulty individual cognitive processes may instead stem from environmental factors—such as unprecedented clinical situations, poor doctor-patient relationships, malfunctioning equipment, work overload, or fatigue. Thus, acknowledging this ecological dimension of clinical reasoning is crucial, both for improving medical decision-making and designing effective educational and institutional interventions. For instance, while extraordinary unsafe environments may be difficult to anticipate or avoid, ordinary unsafe environments—such as those involving communication breakdowns or chronic understaffing—can often be recognized and mitigated. In the former case, educational programs aimed at improving doctor-patient communication and trust are essential; in the latter, institutional reforms are needed to alleviate workload pressures in emergency departments and hospital settings more broadly.

Third, the role and impact of specific cognitive biases are likely to vary across different medical specialties. For instance, *premature closure* may operate differently in paediatrics compared to geriatrics, where possible comorbidities, symptom presentation, and diagnostic urgency differ significantly. Further comparative research is therefore needed to clarify how cognitive biases manifest and interact across various domains of medical practice.

In sum, rather than merely seeing cognitive bias as an obstacle to eliminate *sic et simpliciter*, we should treat it as a context-sensitive feature of human reasoning—sometimes harmful, sometimes helpful, and often complex. The goal is not simple neutrality, but instead discernment: knowing when cognitive bias impairs judgment—and when it enables reliable diagnoses and good care.
